# dAtaxin-2 Mediates Expanded Ataxin-1-Induced Neurodegeneration in a *Drosophila* Model of SCA1

**DOI:** 10.1371/journal.pgen.0030234

**Published:** 2007-12-28

**Authors:** Ismael Al-Ramahi, Alma M Pérez, Janghoo Lim, Minghang Zhang, Rie Sorensen, Maria de Haro, Joana Branco, Stefan M Pulst, Huda Y Zoghbi, Juan Botas

**Affiliations:** 1 Department of Molecular and Human Genetics, Baylor College of Medicine, Houston, Texas, United States of America; 2 Departamento de Biología, Facultad de Ciencias-University Autonoma de Madrid, Madrid, Spain; 3 Division of Neurology, Cedars-Sinai Medical Center; Departments of Medicine and Neurobiology, David Geffen School of Medicine, University of California at Los Angeles, Los Angeles, California, United States of America; 4 Department of Neuroscience, Baylor College of Medicine, Houston, Texas, United States of America; 5 Howard Hughes Medical Institute, Baylor College of Medicine, Houston, Texas, United States of America; University of Minnesota, United States of America

## Abstract

Spinocerebellar ataxias (SCAs) are a genetically heterogeneous group of neurodegenerative disorders sharing atrophy of the cerebellum as a common feature. SCA1 and SCA2 are two ataxias caused by expansion of polyglutamine tracts in Ataxin-1 (ATXN1) and Ataxin-2 (ATXN2), respectively, two proteins that are otherwise unrelated. Here, we use a *Drosophila* model of SCA1 to unveil molecular mechanisms linking Ataxin-1 with Ataxin-2 during SCA1 pathogenesis. We show that wild-type *Drosophila Ataxin-2* (*dAtx2*) is a major genetic modifier of human expanded Ataxin-1 (Ataxin-1[82Q]) toxicity. Increased dAtx2 levels enhance, and more importantly, decreased dAtx2 levels suppress Ataxin-1[82Q]-induced neurodegeneration, thereby ruling out a pathogenic mechanism by depletion of dAtx2. Although Ataxin-2 is normally cytoplasmic and Ataxin-1 nuclear, we show that both dAtx2 and hAtaxin-2 physically interact with Ataxin-1. Furthermore, we show that expanded Ataxin-1 induces intranuclear accumulation of dAtx2/hAtaxin-2 in both *Drosophila* and SCA1 postmortem neurons. These observations suggest that nuclear accumulation of Ataxin-2 contributes to expanded Ataxin-1-induced toxicity. We tested this hypothesis engineering *dAtx2* transgenes with nuclear localization signal (NLS) and nuclear export signal (NES). We find that NLS-dAtx2, but not NES-dAtx2, mimics the neurodegenerative phenotypes caused by Ataxin-1[82Q], including repression of the proneural factor Senseless. Altogether, these findings reveal a previously unknown functional link between neurodegenerative disorders with common clinical features but different etiology.

## Author Summary

The spinocerebellar ataxias (SCAs) are a group of ∼30 neurodegenerative disorders caused by different types of mutations in a variety of unrelated genes. For example, SCA1 and SCA2 are caused by mutations in Ataxin-1 and Ataxin-2, two proteins related in name only. Despite these differences, most SCAs share a number of striking clinical and neuropathological similarities, such as ataxia, tremor, speech difficulties, and atrophy of the cerebellum and brainstem. In addition, many ataxia-causing proteins share interacting protein partners. Together, these observations suggest that many SCAs also share common mechanisms of pathogenesis. Here, we report previously unknown functional interactions between the genes and proteins responsible for SCA1 and SCA2. We find that Ataxin-1 and Ataxin-2 physically interact, and that mutant Ataxin-1 forces Ataxin-2 to accumulate in the nucleus instead of the cytoplasm. Most importantly, using an animal model, we discovered that the *Drosophila* Ataxin-2 gene is a strong suppressor of Ataxin-1-induced neurotoxicity. Thus, neuronal degeneration may take place through common mechanisms in different SCAs. These findings open the possibility of future common therapies for these neurodegenerative disorders for which there is no effective treatment.

## Introduction

Inherited ataxias are a genetically heterogeneous group of neurodegenerative diseases characterized by loss of motor coordination and balance. They can be caused by loss-of-function or gain-of-function mechanisms; some ataxias are triggered by missense mutations, while others by triplet repeat expansions, which may occur either in coding or non-coding sequences. Furthermore, the gene products implicated in the different ataxias do not share obvious functional or structural relationships to each other. In spite of this genetic heterogeneity, many ataxias show striking similarities. In particular, it is often difficult to distinguish between Spinocerebellar ataxias (SCAs) based only on clinical and pathological observations, and their differential diagnosis often requires genetic testing. In addition, a common neuropathological feature of SCAs is the atrophy of the cerebellar module (reviewed in [1–3]). These similarities suggest that SCAs, and perhaps other ataxias, may also share common mechanisms of pathogenesis. In support of this hypothesis a recent study reported a network of physical protein-protein interactions among many factors associated with ataxia and Purkinje cell degeneration in humans and mice [4]. However, no specific molecular mechanisms are known that can account for the clinical and neuoropathological similarities among SCAs and other ataxias.

SCA1 is caused by the expansion of a CAG repeat encoding a polyglutamine tract in the protein Ataxin-1 that induces a toxic gain of function [5]. The expanded protein accumulates in neuronal nuclear inclusions (NIs) that also contain transcription factors, chaperones, proteasome subunits, and other components of the protein quality control/degradation machinery like CHIP or Ataxin-3 [6–11]. Abnormally long polyglutamine tracts are the common cause of pathogenesis in at least five other SCAs (SCA2, 3, 6, 7 and 17) and three additional neurodegenerative diseases including Huntington's disease (HD) [1,12]. Protein quality control machinery as well as transcriptional dysregulation are general mechanisms that have been implicated in the pathogenesis of these polyglutamine disorders [13–15].

Although the polyglutamine expansion triggers the toxicity of Ataxin-1, experiments in *Drosophila* and mouse SCA1 models have shown that protein context plays a key role in expanded Ataxin-1-induced neurodegeneration (reviewed in [15]). The nuclear localization signal[16] and phosphorylation[17] influence the toxicity of expanded Ataxin-1. In addition, certain interacting partners of unexpanded Ataxin-1 are critical to expanded Ataxin-1 toxicity [9,18,19]. In this context, expanded Ataxin-1 was recently found to induce a decrease in the levels of Senseless (Sens) and its murine orthologue growth factor independent 1 (Gfi1) [18]. These are transcription factors that interact with unexpanded Ataxin-1 and are necessary for Purkinje cell survival in mice [18] and for sensory organ development in *Drosophila* [20]. The importance of the protein framework has also been shown in models of other polyglutamine diseases [15,21].

Genetic screening in *Drosophila* models of neurodegenerative diseases is a powerful approach to identify modifier genes and pathways implicated in pathogenesis [22–24]. We previously reported an unbiased genetic screen with a *Drosophila* model of SCA1 [25]. Here we report the identification of the *Drosophila* homolog of Ataxin-2 (*dAtx2*) as a major modifier of expanded Ataxin-1-induced toxicity. Ataxin-2 is a widely expressed cytoplasmic protein with no similarity to Ataxin-1 except for the polyglutamine domain. The normal function of Ataxin-2 remains unclear, although it has been implicated in mRNA processing [26–28] and translational regulation in yeast [29,30], C. elegans[31] and *Drosophila*[32], where it is also required for actin filament formation [33]. However, expansion of its polyglutamine domain leads to SCA2 [34–36]. The functional interactions between Ataxin-1 and Ataxin-2 described here mechanistically tie these two proteins and point to previously unknown pathogenic links between two inherited ataxias.

## Results

### Increased/Reduced Levels of Ataxin-2 Enhance/Suppress Expanded Ataxin-1 Toxicity in the *Drosophila* Eye

**Figure 1:**
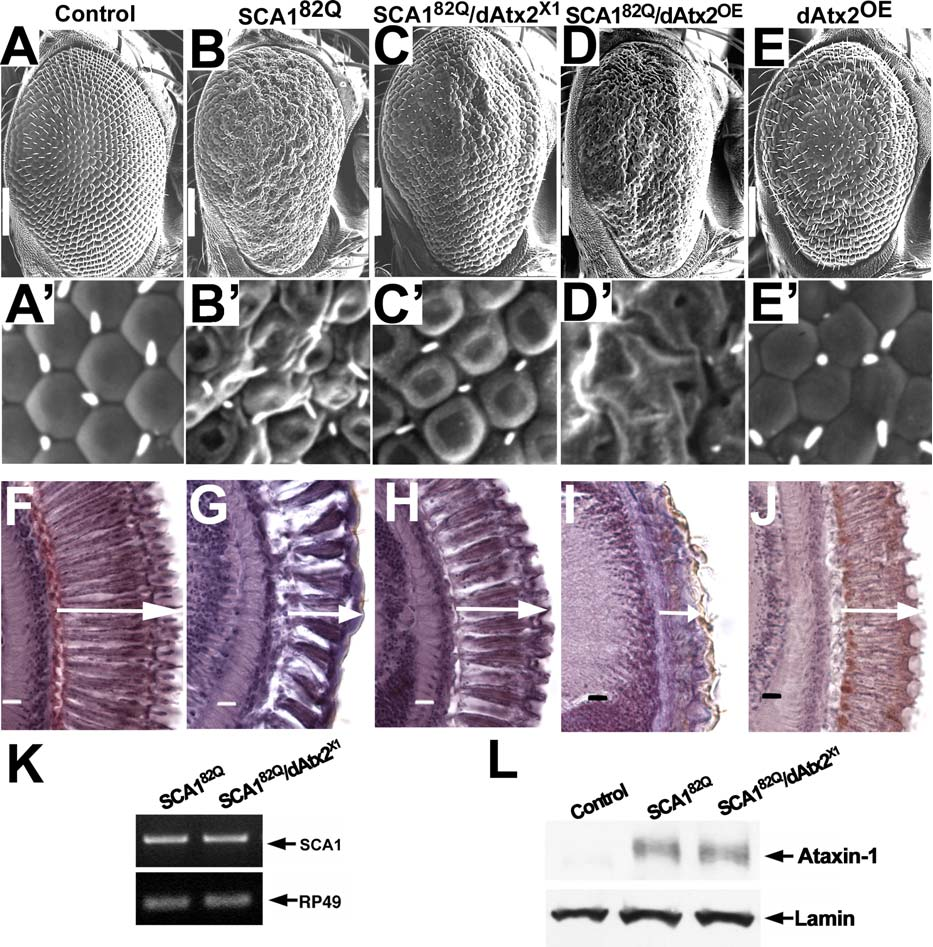
dAtx2 Levels Modulate Ataxin-1[82Q]-Induced Eye Neurotoxicity (A–E, A′–E′) Scanning electron microscopy (SEM) eye images and (F–J) retinal paraffin sections of eyes from the genotype combinations indicated on the top. Arrows indicate photoreceptor length. Transgenes are expressed form the *gmr-GAL4* eye driver. (A, F) Control eyes show regularly arranged ommatidia, and evenly distributed interommatidial bristles (A). Control retinas have long, straight photoreceptors with no gaps (F). (B, G) *SCA1^82Q^* eyes have disorganized ommatidia and interommatidial bristles are unevenly distributed or missing (compare B and A). *SCA1^82Q^* retinas show fewer photoreceptors that are shortened and curved (compare G with F). (C, H) Suppression of external and retinal degeneration in *SCA1^82Q^* flies with decreased dAtx2 levels (*SCA1^82Q^/dAtx2^X1^*). Note the regular arrangement of the ommatidia in *SCA1^82Q^/dAtx2^X1^* animals when compared to *SCA1^82Q^* eyes with normal levels of dAtx2 (compare C with B). Note also the improved retinal organization with longer straight photoreceptors (compare H with G). (D, I) Eyes co-expressing Ataxin-1[82Q] and wild type dAtx2 (*SCA1^82Q^/dAtx2^OE^*) have more disorganized ommatidia when compared to *SCA1^82Q^* alone. Note the absence of bristles (compare D with B). In addition, photoreceptors are shorter and more degenerated (compare I with H). (E, J) Eyes overexpressing low levels of dAtx2 (*dAtx2^OE^*) show mild ommatidial disorganization, with few missing bristles (compare E with A). Internally, photoreceptor cells are shortened (compare J with F). A-E flies were raised at 27°C and F-J at 25°C. (K) Semiquantitative RT-PCR revealing the levels of *SCA1^82Q^* messenger RNA in flies with different levels of dAtx2. (L) Western blot analysis revealing the levels of Ataxin-1[82Q] protein in flies with different levels of dAtx2. Genotypes: (A–J): Control: *yw/+; gmr-GAL4/UAS-LacZ*. SCA1^82Q^: *UAS- SCA1^82Q^ [F7]/yw; gmr-GAL4/+*. SCA1^82Q^/dAtx2^X1^: *SCA1^82Q^ [F7]/yw; gmr-GAL4/+; dAtx2^X1^*/+. SCA1^82Q^/ dAtx2^OE^: *UAS-SCA1^82Q^ [F7]/yw; gmr-GAL4/UAS- dAtx2 [4].* dAtx2^OE^: *gmr-GAL4/UAS- dAtx2 [4]*. (A–E) SEM scale bar=100μm. (A′–E′) are 30μm X 30μm. (F–J) scale bar=10μm.

Expression of Ataxin-1[82Q] in the eye of *SCA1^82Q^* flies causes external and internal abnormal phenotypes [25]. Externally, the eyes of these animals show severe ommatidial disorganization as well as interommatidial bristle loss when compared with control eyes (Figure 1, compare A and A′ with B and B′). Internally, examination of the retina reveals tissue loss and shortened and curved photoreceptor neurons (Figure 1, compare F with G). In a screen for genetic modifiers of Ataxin-1[82Q]-induced toxicity we recovered *EP(3)3145* as an enhancer of the eye phenotype (data not shown). This is an insertion of an EP transposable element [37] in the 5′ end of *dAtx2*, the *Drosophila* orthologue of human Ataxin-2. The *Drosophila* and human proteins share 23% amino acid identity and 36% amino-acid similarity over the entire protein with the most conserved sequences corresponding to the ATX2-N and ATX2-C domains (43% and 62% identity, respectively) [33]. Molecular analysis revealed that the EP element is inserted 3121 bp upstream of the ATG and in the same orientation as the *dAtx2* transcription unit (data not shown and [33]). These data suggested that *EP(3)3145* over-expresses the *dAtx2* transcription unit to enhance the *SCA1^82Q^* eye phenotype. As described below, this possibility was confirmed using a transgene that over-expresses the *dAtx2* cDNA.

Co-expression of a wild-type dAtx2 transgene (*dAtx2^OE^*) at low levels enhances the Ataxin-1[82Q]-induced eye phenotype. Externally, the eyes of *SCA1^82Q^/dAtx2^OE^* animals show no bristles and increased ommatidial disorganization when compared with the eyes of *SCA1^82Q^* controls (compare Figure 1D and D′ with B and B′). Internally, photoreceptor cells are considerably shorter (compare Figure 1I with G). Expression of the same low levels of dAtx2 alone in the eye causes relatively mild external disorganization and reduction of the retinal width (Figure 1E, E′ and J). Overexpression of dAtx2 from *EP(3)3145* and *UAS-dAtx2* also aggravates the phenotypes of other fly models of neurodegenerative diseases besides SCA1 [38,39]. However, since overexpression of dAtx2 causes an eye phenotype by itself (Figure 1E, E′ and J) and it is toxic in many other tissues [33], it is difficult to make strong conclusions about the specificity of these genetic interactions.

To test the specificity of the genetic interaction, we investigated if decreasing the levels of endogenous dAtx2 modifies expanded Ataxin-1-induced toxicity. For this, we used a 1.4 kb deletion in the *dAtx2* locus (*dAtx2^X1^*) that removes part of the *dAtx2* promoter, the ATG codon and extends into the first intron [33]. We find that flies expressing Ataxin-1[82Q] and heterozygous for the *dAtx2^X1^* mutant allele show a strong suppression of the eye phenotype, with much improved arrangement of the ommatidia and bristles compared to eyes from flies expressing Ataxin-1[82Q] with normal dAtx2 levels (compare Figure 1C and C′ with B and B′). This suppression is also evident in the retinas of *SCA1^82Q^/dAtx2^X1^* flies that show elongated photoreceptors and very little tissue loss (compare Figure 1H with G). To further test the specificity of this interaction, and to exclude potential genetic background artefacts, we asked whether adding back dAtx2 to *SCA1^82Q^/dAtx2^X1^* flies eliminates the suppression effect. Figure S1 shows that *SCA1^82Q^/dAtx2^X1^*/*dAtx2^OE^* flies show an eye phenotype that is very similar to the phenotype of *SCA1^82Q^* flies. The effects of the *dAtx2^X1^* and *dAtx2^OE^* alleles decreasing/increasing dAtx2 levels are demonstrated in Figure S2.

Since dAtx2 is an RNA binding protein, we investigated if the observed suppression of Ataxin-1[82Q] toxicity was the result of dAtx2 affecting the levels of the *SCA1^82Q^* mRNA transcript or the levels of Ataxin-1 [82Q] protein. As shown in Figure 1K and L neither the levels of *SCA1^82Q^* mRNA nor the levels of the Ataxin-1[82Q] protein are affected by changing the levels of dAtx2.

### Reduced Levels of Ataxin-2 Suppress Expanded Ataxin-1-Induced Neuronal Dysfunction in *Drosophila*

To verify that the genetic interaction between Ataxin-1 and dAtx2 is not limited to the eye, we analyzed the effect of altering dAtx2 levels on expanded Ataxin-1-induced neuronal dysfunction.

**Figure 2:**
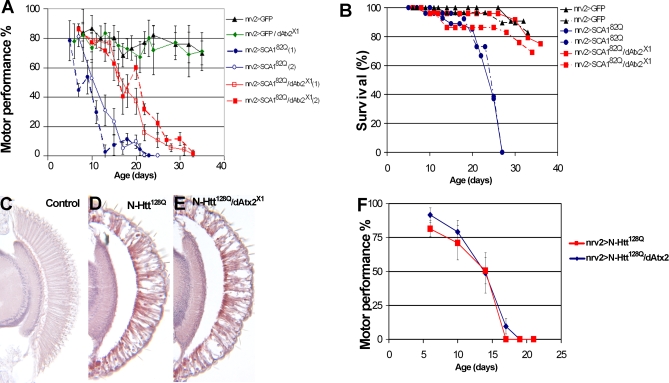
Specificity of the dAtx2/ Ataxin-1 Interaction (A, B) Reduced levels of dAtx2 also suppress Ataxin-1[82Q]-induced motor dysfunction and shortened life span. (A) Motor performance quantification in flies of the indicated genotypes measured as climbing ability as a function of age. Control animals expressing just GFP perform well in the motor assay for 36 days (*nrv2>GFP*, black triangles), as do flies that are heterozygous for the *dAtx2^X1^* allele (*nrv2>GFP/dAtx2^X1^*, green diamonds). Flies expressing Ataxin-1[82Q] in the nervous system show progressive motor dysfunction when compared with controls (*nrv2> SCA1^82Q^*, blue circles). All *nrv2> SCA1^82Q^* flies stop climbing after ∼23 days. Flies expressing Ataxin-1[82Q] in the nervous system but with decreased dAtx2 levels show improved motor performance (*nrv2> SCA1^82Q^/dAtx2^X1^*, red squares). Error bars represent standard deviation. Flies were raised at 27°C. (B) Survivorship in flies of indicated genotypes. Control flies (*nrv2>GFP*, black triangles) do not show significant mortality during the first 30 days (*nrv2>GFP*, black triangles). Expression of Ataxin-1 [82Q] in the nervous system leads to early mortality (*nrv2> SCA1^82Q^*, blue circles). This phenotype is suppressed by decreasing the levels of dAtx2 (*nrv2> SCA1^82Q^/dAtx2^X1^*, red squares). (C–F) Reduced levels of dAtx2 do not suppress eye neurotoxicity or motor impairments caused by N-Htt^128Q^. (C–E) Expression of N-Htt^128Q^ in the eye leads to retinal degeneration and loss of photoreceptors and other cell types (compare D with control shown in C). This eye phenotype is not altered in animals with reduced levels of dAtx2 (compare D and E). (F) Expression of N-Htt^128Q^ in the nervous system leads to progressive motor dysfunction (*nrv2> N-Htt^128Q^*, red), and decreasing the levels of dAtx2 does not significantly suppress this phenotype (*nrv2> N-Htt^128Q^/dAtx2^X1^*, blue) Genotypes: (A–B): nrv2>GFP: *nrv2-GAL4/UAS-eGFP*. nrv2>GFP/dAtx2^X1^: *nrv2-GAL4/UAS-eGFP; dAtx2^X1^/+.* nrv2> SCA1^82Q^: *nrv2-GAL4/+; UAS-SCA1^82Q^ [M6]/+*. nrv2> SCA1^82Q^/dAtx2^X1^: *nrv2-GAL4/+; UAS-SCA1^82Q^ [M6]/dAtx2^X1^*. (C) *yw; gmr-GAL4/UAS-GFP*. (D) *yw; gmr-GAL4/+; UAS-N-Htt^128Q^/+*. (E) *yw; gmr-GAL4/+; UAS-N-Htt^128Q^/dAtx2^X1^*. (F) nrv2>N-Htt^128Q^: *nrv2-GAL4/+; UAS-N-Htt^128Q^/+.* nrv2>N-Htt^128Q^/dAtx2^X1^: *nrv2-GAL4/*+; *UAS-N-Htt^128Q^/ dAtx2^X1^*.

The motor performance of flies as a function of age can be quantified using a climbing assay [40]. This assay has been used to analyze the effects of toxic proteins on neurons in other *Drosophila* models of neurodegenerative diseases [41,42]. Control flies show no significant decrease in their motor performance until late in life. Figure 2A shows that ∼74% of control flies still climb after thirty-six days (black triangles). Flies expressing Ataxin-1[82Q] specifically in the nervous system (using *nrv2-GAL4*) display a progressive impairment of their motor performance (Figure 2A, blue circles). In the context of the *Drosophila* life span, this is a late onset and progressive phenotype as compared to the performance of control flies in the same period of time. We then analyzed the effect of decreased levels of dAtx2 on the climbing phenotype caused by Ataxin-1[82Q]. As shown in Figure 2A (red squares) in *SCA1^82Q^* flies also heterozygous for the *dAtx2^X1^* mutation climbing performance is significantly improved compared to flies with normal dAtx2 levels (p<0.0001 for repeated measures anova (rma) between genotypes). Figure 2A show that while all *SCA1^82Q^* animals fail to climb after 26 days, *SCA1^82Q^/ dAtx2^X1^* flies continue to climb until later in life. Thus the impairments in motor performance caused by neuronal expression of Ataxin-1[82Q] are suppressed by decreased *dAtx2* levels.

We also studied the effect of Ataxin-1[82Q] expression in a life span assay. Figure 2B shows that expression of Ataxin-1[82Q] in the nervous system leads to premature death in *SCA1^82Q^* flies in comparison to *GFP* controls (Figure 2B, compare blue circles with black triangles). While *SCA1^82Q^* animals do not survive past 30 days, this early lethality phenotype is suppressed in *SCA1^82Q^/ dAtx2^X1^*animals (Figure 2B, red squares).

### Decreased Levels of dAtx2 Do Not Suppress Neurodegeneration Caused by Expanded Huntingtin

To investigate whether dAtx-2 also modulates neurodegeneration in other models of polyglutamine disease, we tested the effect of altering the dAtx2 levels in a *Drosophila* model of Huntington's disease [7,43]. Adult flies expressing an expanded N-terminal fragment of human huntingtin (N-Htt^128Q^) in the eye show a progressive retinal degeneration which becomes obvious at day 5 after eclosion [7]. N-Htt^128Q^ retinas show disorganized and missing photoreceptors (Figure 2D, compare with control in 2C). *N-Htt^128Q^* flies also overexpressing dAtx2 present a more degenerated retina than flies expressing N-Htt^128Q^ with normal levels of dAtx2 (data not shown). However, since overexpression of dAtx2 is sufficient to cause retinal degeneration (Figure 1J), this result is not conclusive by itself. Therefore, we tested the effect of decreasing dAtx2 levels on the N-Htt^128Q^ induced retinal degeneration. As shown in Figure 2D-E, decreasing the levels of dAtx2 (*N-Htt^128Q^/dAtx2^X1^*) does not obviously alter degeneration induced by N-Htt^128Q^ in the *Drosophila* eye.

We also investigated a possible genetic interaction between dAtx2 and N-Htt^128Q^ in the motor performance assay described above. Like with Ataxin-1[82Q], expression of N-Htt^128Q^ in the nervous system leads to motor performance impairments (Figure 2F) where N-Htt^128Q^ animals stop climbing before day 20. The climbing performance of animals expressing N-Htt^128Q^ with decreased levels of dAtx2 (*N-Htt^128Q^/dAtx2^X1^*) is not significantly different from that of animals expressing N-Htt^128Q^ with normal levels of dAtx2 (Figure 2F). Therefore decreasing the levels of dAtx2 fails to suppress N-Htt^128Q^ induced degeneration both in the retina and in the nervous system.

### Ataxin-2 Levels Modulate Expanded Ataxin-1-Induced Loss of Mechanoreceptors Caused by Loss of Senseless Protein

Senseless (Sens) is a proneural factor that is expressed and required in the sensory organ precursor (SOP) cells of the peripheral nervous system [20]. Expression of high levels of Ataxin-1[82Q] in the thoracic SOPs using *scabrous-GAL4* (*sca-GAL4*) leads to a reduction in Sens protein levels in these cells and loss of large mechanoreceptors (macrochaetae) in the thorax of adult flies [18]. Therefore, scoring adult thoracic macrochaetae in *SCA1^82Q^* animals provides a quantitative phenotype with a known molecular foundation.

**Figure 3:**
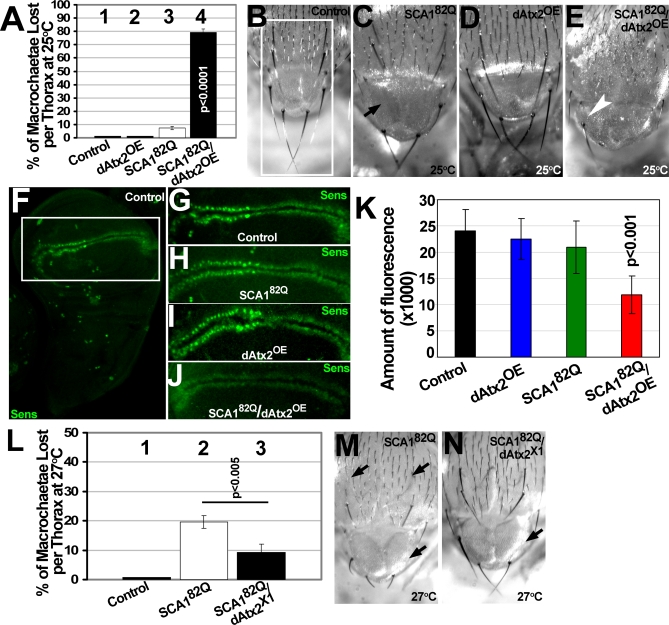
dAtx2 Levels Modulate Ataxin-1[82Q]-Induced Loss of Mechanoreceptors (A) Percentage of missing thoracic macrochaetae (large mechanoreceptor bristles) over a total of 26 in flies of the indicated genotypes raised at 25°C. Columns-1 and 2, no macrochaetae loss is detected in control flies or flies over-expressing dAtx2 (*dAtx2^OE^*) in the thoracic sensory organ precursor (SOP) cells respectively with the *scabrous-GAL4* driver. Column-3, mild decrease in thoracic macrochaetae number caused by expression of Ataxin-1[82Q] at low levels in the SOP cells (p<0.0001). Column-4, ∼80% decrease in the number of thoracic macrochaetae in animals co-expressing Ataxin-1[82Q] and dAtx2 (*SCA1^82Q^/dAtx2^OE^*) in the SOPs (p<0.0001). Error bars=s.e.m. (B–E) Images of the macrochaetae present in the posterior region of the *notum* and *scutellum* (white rectangle in B) of flies with same genotypes as (A). (B) Control thoraxes have eight macrochaetae in the selected area (white rectangle). (C-E) Over-expression of dAtx2 enhances Ataxin-1[82Q] induced macrochaetae loss. Black arrow indicates missing bristle in C and white arrow in E points to the only bristle present in the *scutellum* of an *SCA1^82Q^/dAtx2^OE^* animal. Flies grown at 25°C. (F) Anti-Sens immunofluorescence revealing the normal pattern of Sens distribution in the wing disc. White rectangle highlights the wing margin SOP and bristle precursor cells. (G–J) Close-up of the wing margin region from: control animals (G), animals expressing Ataxin-1[82Q] alone (H), wild-type dAtx2 (I) or coexpressing Ataxin-1[82Q] and dAtx2 (J). (K) Quantification of anti-Sens signal in the wing margin of animals of the genotypes indicated on the bottom. 20 wing discs were used per genotype and experiments carried out at 25°C (see materials and methods for more details). Bars=Std. Dev. (L) Percentage of lost macrochaetae in flies expressing Ataxin-1[82Q] with normal or decreased levels of dAtx2. Flies grown at 27°C to boost expression levels of the *GAL4/UAS* system. Column-1, control animals do not show macrochaetae loss. Column-2, ∼20% of thoracic macrochaetae are lost after expression of Ataxin-1[82Q] at high levels in the SOP cells (p<0.0001). Column-3, partial rescue of Ataxin-1[82Q]-induced macrochaetae loss in animals heterozygous for the *dAtx2^X1^* mutant (p<0.005). Bars= s.e.m. (M–N) Images of the macrochaetae present in the posterior *notum* and *scutellum* (white rectangle in B) of flies with same genotypes as L. Arrows indicate missing bristles. Flies grown at 27°C. Genotypes: control: *sca^109–68^-GAL4/UAS-GFP*. dAtx2^OE^:*sca^109–68^-GAL4/ UAS- dAtx2 [4]*. SCA1^82Q^: *UAS- SCA1^82Q^ [F7]/+; sca^109–68^-GAL4/+*. SCA1^82Q^/dAtx2^OE^: *UAS- SCA1^82Q^ [F7]/+; sca^109–68^-GAL4/ UAS- dAtx2 [4]*. SCA1^82Q^/dAtx2^X1^: *UAS-SCA1^82Q^ [F7]/+; sca^109–68^-GAL4/+; dAtx2^X1^*/+.

We analyzed the effect of altering Ataxin-2 levels on Ataxin-1[82Q]-induced loss of mechanoreceptors. This was performed by quantifying the number of macrochaetae in the adult thorax of *SCA1^82Q^* animals with different levels of dAtx2. Using a relatively low expressing Ataxin-1[82Q] line, we defined conditions in which *sca-GAL4*-mediated expression in the SOP cells causes only 9% of macrochaetae loss (Figure 3A-column-3 and Fig 3C, compare to controls Figure 3A-column-1 and Figure 3B). Expression of wild-type dAtx2 alone (*dAtx2^OE^*) leads to no loss of machrochaetae (Figure 3A-column-2 and Figure 3D), but co-expression of dAtx2 and Ataxin-1[82Q] (*SCA1^82Q^/dAtx2^OE^*) leads to a severe loss of macrochaetae compared to Ataxin-1[82Q] alone (Figure 3E, compare with Figure 3C). Quantification shows an ∼80% decrease in the number of macrochaetae when both proteins are co-expressed compared to controls (Figure 3A-columns 1–4, p<0.0001, Tukey-Kramer HSD).

In the wing imaginal disc, Sens expression includes the precursors of the large thoracic mechanoreceptor bristles, and the two parallel rows of bristles at each side of the wing margin (Figs. 3F-G). We quantified the amount of fluorescence detected after anti-Sens immunostaining in wing margin cells expressing Ataxin-1[82Q] with normal or increased levels of dAtx2. Expression of either low levels of Ataxin-1[82Q] or dAtx2 with *sca-Gal4* (at 25C) produces no detectable decrease in the levels of Sens in the wing margin (Figure 3H and I compare with G and quantification in K). However co-expression of both Ataxin-1[82Q] and dAtx2 in the same conditions induces a strong decrease in the levels of Sens (Figure 3J). Quantification of the Sens signal in *SCA1^82Q^/dAtx2^OE^* animals (Figure 3K, fourth column) reveals a decrease of ∼50% in the amount of Sens signal when compared to either wild type, *SCA1^82Q^* or *dAtx2^OE^* controls (p<0.001, Tukey-Kramer HSD).

In addition, we investigated the consequences of decreasing the amount of dAtx2 (using the *dAtx2^X1^* allele) in conditions where Ataxin-1[82Q] reduces Sens levels in the wing margin (i.e. at 27°C and 29°C). However, we did not detect a significant modification (data not shown). Since it is possible that we are not able to detect small changes on Sens levels in wing discs from late third instar larvae, or this changes might happen later in the SOP development, we also investigated the effect of decreasing the amount of Ataxin-2 on Ataxin-1[82Q]-induced mechanoreceptor loss. Expression of Ataxin-1[82Q] at high levels (27°C) in the SOP cells results in the loss of ∼20% of thoracic macrochaetae when compared to controls (Figure 3L compare columns 1 and 2 p<0.0001, Tukey-Kramer HSD; see Figure 3M). Reducing the levels of endogenous dAtx2 with the heterozygous *dAtx2^X1^* mutation partially rescues the Ataxin-1[82Q]-induced bristle phenotype. Loss of macrochaetae in *SCA1^82Q^/ dAtx2^X1^* animals is approximately half of that seen in animals with normal levels Ataxin-2 (Figure 3L, compare columns 2 and 3 p<0.005, Tukey-Kramer HSD, and see Figure 3N).

### Physical Interaction between Human Expanded Ataxin-1 and Ataxin-2 Proteins

**Figure 4:**
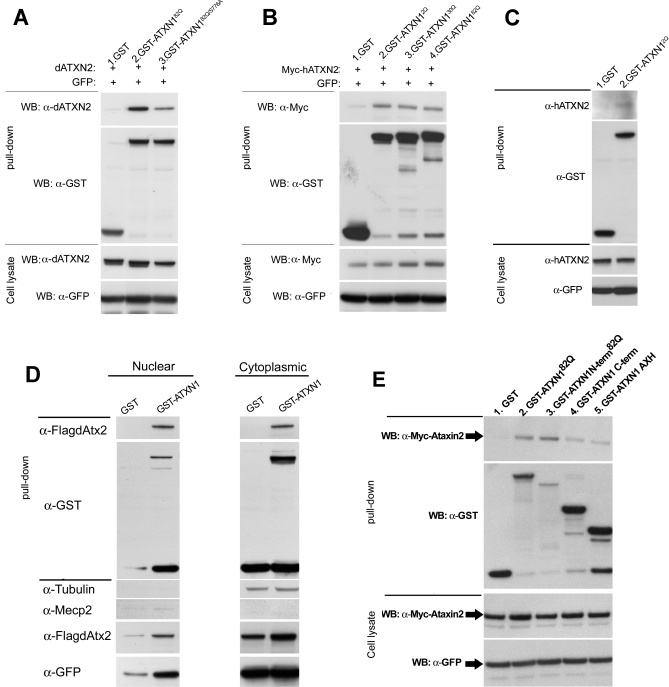
The Ataxin-1[82Q] and Ataxin-2 Proteins Physically Interact (A) GST Co-AP pull-down experiments between dAtx2 and GST-constructs carrying human Ataxin-1[82Q] or Ataxin-1[82Q] with S776A mutation. (B) GST Co-AP experiments between human Ataxin-2 (Myc-hATXN2) and GST-Ataxin-1 with different polyglutamine lengths. (C) Interaction between GST-Ataxin-1[2Q] and endogenous hATXN2 in cell culture. (D) Co-AP pull down experiments between GST-Ataxin-1 and Flag-dAtx2 after nuclear/cytoplasmic fractionation. Tubulin is used as cytoplasmic marker and Mecp2 as nuclear marker. (E) Comparative analysis of the interaction of hAtaxin-2 with different domains of the Ataxin-1 protein. Lane-1, GST alone does not pull down Myc-hAtaxin2. Lane-2, GST-ATXN1^82Q^ pulls down Myc-hAtaxin2. Lane-3 expanded N-terminal Ataxin-1 (GST-ATXN1N-term^82Q^) lacking the AXH domain (aa# 1–575) also pulls down Myc-hAtaxin-2 with high affinity. Lanes-4 and 5 show that both the C-terminus portion of Ataxin-1 containing the AXH domain (aa# 529–816; lane-4) or the AXH domain alone (aa# 558–700; lane-5) pull down Myc-hAtaxin2. These interactions are weaker than that of the N-terminus part (compare lanes 4 and 5 with lane 3). Cell lysates for co-AP were ran through Glutathione conjugated beads, and blots stained with anti-GST or anti-Myc. Immunoblots for dAtx2, Myc-hAtaxin2 and GFP carried out on cell lysates before the co-AP pull-down reveal similar levels of both proteins between samples.

To further characterize the interactions between Ataxin-1 and Ataxin-2, we investigated possible protein-protein interactions. Lysates from cells expressing *Drosophila* or human Ataxin-2 and GST-Ataxin-1 [82Q] were subjected to co-affinity purification (co-AP) glutathione-S-transferase (GST) pull-down assays. As shown in Figure 4A (lanes 1 and 2) and Figure 4B (lanes 1 and 4), GST-Ataxin-1[82Q] is able to pull down the *Drosophila* and human Ataxin-2 proteins, which indicates that both proteins are able to physically interact. We also asked whether this interaction is polyglutamine dependent. As shown in Figure 4B lanes 2–4 we did not detect significant differences in the interactions between GST-Ataxin-1 with 2, 30 or 82 glutamines and human Ataxin-2 in this co-AP assay.

Ataxin-1[82Q] is phosphorylated at Serine residue 776 on the C-terminal portion of the protein, and a (Ser776Ala) mutation inhibits the toxicity of Ataxin-1[82Q] in mice[17]. We investigated the importance of Ser776 for the Ataxin-1[82Q]-dAtx2 interaction. As shown in (Figure 4A lane 3) the interaction of dAtx2 with Ataxin-1[82Q]^S776A^ is weaker than with normal Ataxin-1[82Q], suggesting that this interaction is phosphorylation dependent.

In addition, we investigated if Ataxin-1 can pull down endogenous hATX2. We find that unexpanded Ataxin-1 is able to precipitate endogenous hATX2 from human cells, suggesting that the two proteins may be functional interactors in vivo (Figure 4C).

We also investigated whether the interaction between Ataxin-1 and Ataxin-2 is cytoplasmic or nuclear. We carried out co-AP assays with Ataxin-1 and dAtx2 after nuclear/cytoplasmic fractionation of cultured cells. Figure 4D shows that we were not able to detect differences in protein interactions between these cellular compartments using this assay.

Lastly, we investigated whether specific domains of the hAtaxin-1 protein are responsible for the interaction with hAtaxin-2. Co-AP experiments were carried out with lysates from cells expressing Myc-hAtaxin-2 and one of the following hAtaxin-1 fragments tagged with GST: polyglutamine expanded N-terminal (aa# 1–575, Figure 4E lane-3), C-terminus Ataxin-1 containing the AXH domain (aa# 529–816; Figure 4E lane-4) or the AXH domain alone (aa# 558–700; Figure 4E lane-5). All three fragments pull-down Myc-hAtaxin-2, indicating that each Ataxin-1 fragment can interact independently with hAtaxin-2 (Figure 4E lanes 3–5). However, the interaction of hAtaxin-2 is stronger with the N-terminal Ataxin-1 fragment (Figure 4E, lane-3) as compared to the C-terminal or AXH portions (Figure 4E lanes 4 and 5 respectively), since less N-terminal peptide pulls down more hAtaxin-2.

### Ataxin-2 Accumulates in the Nucleus of Expanded Ataxin-1-Expressing *Drosophila* Cells and Human Neurons

**Figure 5:**
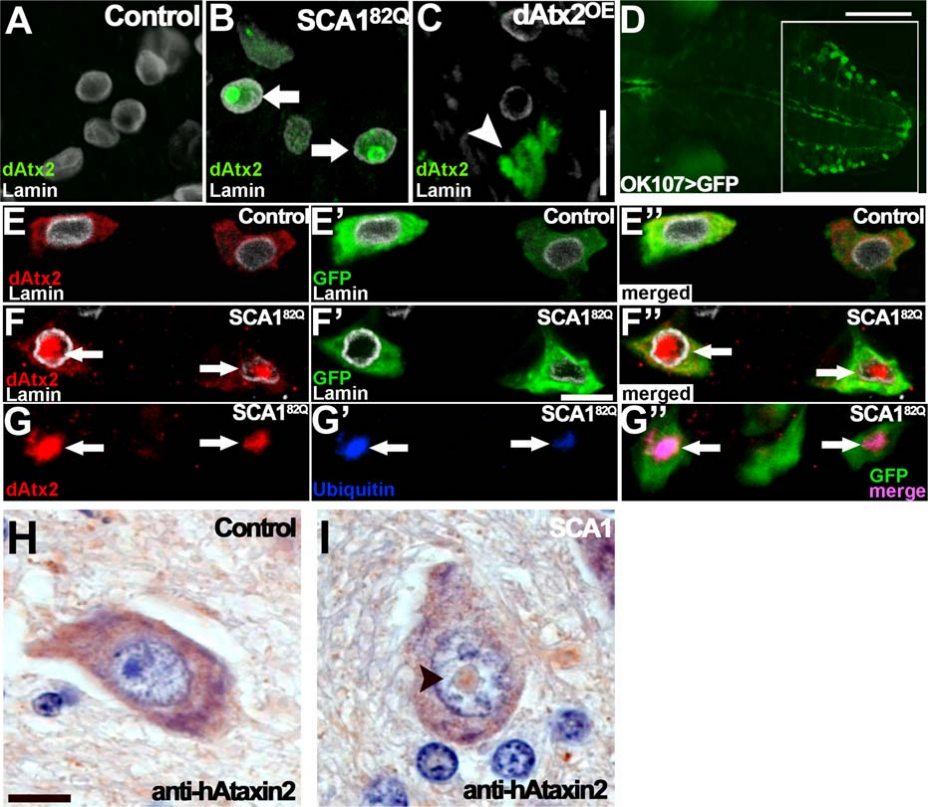
Expanded Ataxin-1 Induces Nuclear Accumulation of Ataxin-2 in *Drosophila* and Human Neurons (A–C) Longitudinal paraffin sections through adult fly retinas stained with anti-dAtx2 antiserum (green). Nuclei are visualized with anti-Lamin (white). Controls expressing only *gmr-GAL4* driver (A) or *gmr-GAL4* driver plus UAS-*dAtx2* (C) show no detectable nuclear dAtx2 staining; arrowhead in (C) points to cytoplasmic accumulation of dAtx2. (B) dAtx2 localizes to the nucleus and accumulates in NIs (arrows) in retinal cells of *SCA1^82Q^* animals. Also notice additional diffuse dAtx2 signal in the nuclei. (D) Pattern of *ok107-GAL4* in the VNC, showing the area we analyzed (white square). (E–E′′) Confocal images of control *Drosophila* neurons in the larval ventral ganglion expressing CD8:GFP from the *ok107-GAL4* neuronal driver and stained with anti-dAtx2 antibody. (E) No dAtx2 staining (red) is detected in the neuronal nucleus. Nuclear Lamin is shown in white. (E′) Same neurons as E showing CD8:GFP to reveal the neuronal cytoplasm. Images are merged in E′′. (F–F′′) Confocal images of *ok107-GAL4* neurons expressing Ataxin-1[82Q] (SCA1^82Q^) stained in parallel to E–E′′. (F) Shows dAtx2 signal (red) inside the nucleus (white) in the form of NIs (arrows), in addition to the cytoplasmic dAtx2 signal. (F′) Shows the same neurons visualized with CD8:GFP showing neuronal cytoplasm (green). Images are merged in F′′. (G–G′′) Triple immunofluorescence for Ubiquitin (blue), dAtx2 (red) and CD8:GFP (green) in *ok107-GAL4* neurons expressing Ataxin-1[82Q]. Notice the overlapping of the dAtx2 (G) and Ubiquitin (G′) signals in the merged image in G′′ (arrows point to nuclear inclusions). (H, I) Human pontine neurons stained with anti-human Ataxin-2 antibody (brown). Hematoxylin (blue) was used for counterstaining. (H) No staining is detected in neuronal nuclei of non SCA1 postmortem tissue. (I) Ataxin-2 accumulates in NI (arrowhead) in a SCA1 neuron. Genotypes: (A) *gmr-GAL4/+*. (B) *UAS-SCA1^82Q^[F7]/+; gmr-GAL4/+*. (C) *gmr-GAL4/UAS-dAtx2[4].* (D, E-E′′) *UAS-CD8:GFP/+; +; ok107-GAL4/+.* (F-F′′ and G-G′′) *UAS-CD8:GFP/+; UAS-SCA1^82Q^ [M6]/+; ok107-GAL4/+.*

The co-AP assays with expanded hAtaxin-1 and hAtaxin-2 indicate that the two proteins are able to interact. However, Ataxin-1 normally localizes to the nucleus in *Drosophila* and many human cell types, while Ataxin-2 is a cytoplasmic protein. To address whether the interaction observed in cultured cells is relevant in vivo, we monitored the localization of Ataxin-2 in *Drosophila* cells expressing Ataxin-1[82Q]. Since Ataxin-1[82Q]-induced toxicity is suppressed by *dAtx2* loss of function in the *Drosophila* eye; we first analyzed the localization of dAtx2 in retinal cells. dAtx2 is not normally detected in the nuclei of retinal cells from either control eyes or eyes overexpressing dAtx2 (Figure 5A and C respectively). In contrast, we find that endogenous dAtx2 localizes to the nuclei of retinal cells expressing Ataxin-1[82Q]. Furthermore, nuclear dAtx2 signal is detected both diffusely in the nucleoplasm as well as in nuclear inclusions (NIs) (Figure 5B). To confirm this unexpected result, we examined the localization of dAtx2 in other Ataxin-1[82Q]-expressing neurons. Similar to the results obtained in the retina, endogenous dAtx2 normally localizes to the cytoplasm and is not detected in the nuclei of neurons from the ventral nerve cord (Figure 5 E-E′′, *ok107-GAL4* pattern shown in D). However, Ataxin-1[82Q]-expressing VNC neurons show nuclear accumulation of dAtx2 (Figure 5 F-F′′). Next we investigated whether dAtx2 and Ataxin-1 colocalize. Co-staining of Ataxin-1 and dAtx2 is not possible since the available antibodies for both proteins were raised in rabbit; however, Ataxin-1 NIs in *Drosophila* neurons are positive for Ubiquitin [25], so we performed a double staining for dAtx2 and Ubiquitin. Figure 5G-G′′ shows that the dAtx2 NIs present in the neurons of *SCA1^82Q^* flies are positive for Ubiquitin. These results indicate that expanded Ataxin-1 causes dAtx2 to localize to the nucleus and suggest that both proteins co-aggregate in NIs.

To validate these results and investigate their relevance for SCA1 pathogenesis, we analyzed the localization of hAtaxin-2 in SCA1 neurons from human postmortem brain samples by anti-Ataxin-2 immunohistochemistry. Pontine neurons from control samples consistently show cytoplasmic localization of hAtaxin-2 (Figure 5H). Pontine neurons from SCA1 brain samples display frequent Ataxin-1 NIs that are clearly visible with Hematoxylin staining. We found that approximately twenty percent of these NIs are positive for hAtaxin-2 (Figure 5I); see also[10]. These findings, together with the co-AP data using human Ataxin-1 and Ataxin-2 proteins, suggest that the Ataxin-1-Ataxin-2 interactions observed in *Drosophila* may be relevant for SCA1 pathology.

### Nuclear Accumulation of Ataxin-2 Is Induced by Pathogenic Ataxin-1[82Q], but Not Unexpanded Ataxin-1[2Q] and Ataxin-1[30Q]

**Figure 6:**
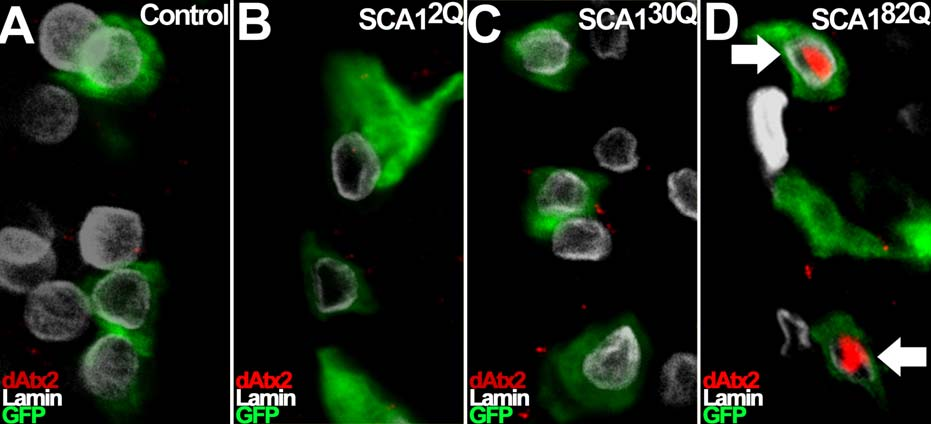
Pathogenic Ataxin-1[82Q], but Not Unexpanded Ataxin-1, Induces Nuclear Accumulation of dAtx2 *In Vivo* (A–D) Anti-dAtx2 immunofluorescence (red) in ventral ganglion neurons expressing different Ataxin-1 transgenes and CD8-GFP (green) with the *ok107-GAL4* driver. Nuclear Lamin (white) is also shown. (A) Control neurons show no nuclear dAtx2. (B) Neurons expressing Ataxin-1 with only 2Q (*SCA1^2Q^*) present no nuclear dAtx2. (C) Neurons expressing wild type human Ataxin-1 with 30Q (*SCA1^30Q^*) fail to show nuclear dAtx2 as well. (D) Neurons expressing expanded Ataxin-1[82Q] (*SCA1^82Q^*) present nuclear dAtx2. Genotypes: (A) *UAS-CD8:GFP/+; +; ok107-GAL4/+*. (B) *UAS-CD8:GFP/+; UAS-SCA1^2Q^ [F14]/+; ok107-GAL4/+* (C) *UAS-SCA1^30Q^ [F1]/yw; UAS-CD8:GFP/+; ok107-GAL4/+*. (D) *UAS-CD8:GFP/+; UAS-SCA1^82Q^ [M6]/+; ok107-GAL4/+*.

SCA1 pathogenesis is triggered by polyglutamine expansion in Ataxin-1 beyond 39–44 residues [5]. To investigate if nuclear accumulation of Ataxin-2 is specific to the pathogenic Ataxin-1 form, we analyzed dAtx2 localization in *Drosophila* VNC neurons expressing human Ataxin-1 with different polyglutamine lengths: Ataxin-1[2Q], Ataxin-1[30Q] and Ataxin-1[82Q]. The Ataxin-1[2Q] line used has higher levels of protein expression than the Ataxin-1[30Q] and [82Q] lines, both of which have comparable expression levels (Western blot data not shown). Figure 6A-D shows that no nuclear dAtx2 signal is detected in control neurons (Figure 6A), or neurons expressing Ataxin-1[2Q] or [30Q] (Figure 6B and C). In contrast, accumulation of dAtx2 is detected in the nucleus of Ataxin-1[82Q]-expressing neurons (Figure 6D). Although this result does not rule out an interaction between wild-type Ataxin-1 and dAtx2, it indicates that abundant nuclear accumulation of Ataxin-2 is specific to the expanded Ataxin-1 form responsible for SCA1 pathogenesis.

### Nuclear Accumulation of Ataxin-2 Causes Severe Eye Toxicity

**Figure 7:**
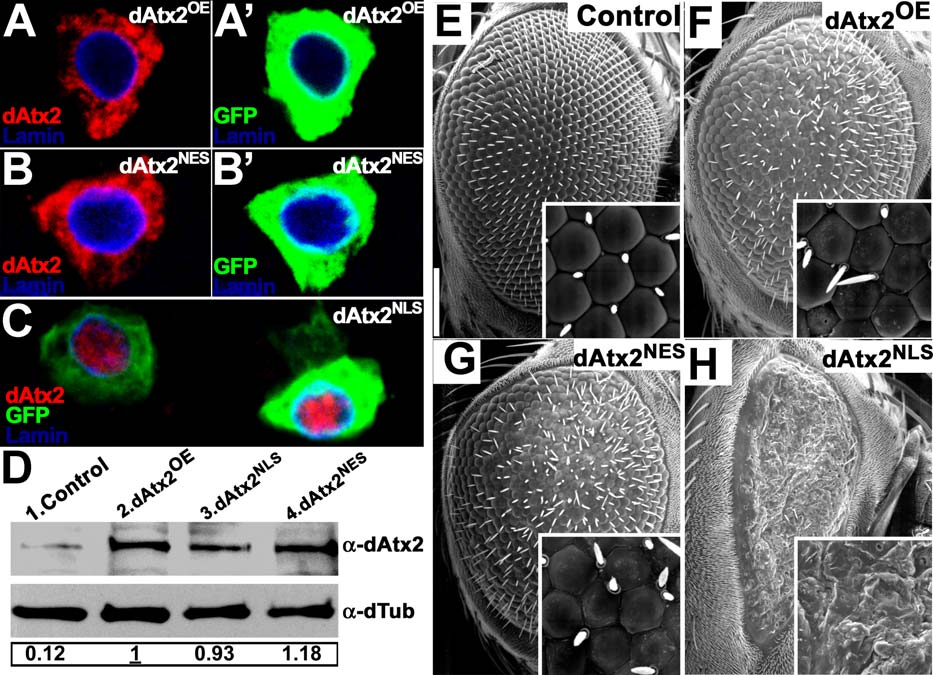
Nuclear Accumulation of dAtx2 Increases Its Toxicity *In Vivo* (A–C) Immunofluorescence staining to reveal the localization of dAtx2 in neurons overexpressing CD8:GFP together with wild-type dAtx2 (dAtx2^OE^) or dAtx2 with a nuclear export or a nuclear localization signal (dAtx2^NES^ or dAtx2^NLS^, respectively). (A and A′) dAtx2 localizes to the cytoplasm even when overexpressed. (B and B′) dAtx2^NES^ distribution is similar to that of wild-type dAtx2. (C) nuclear localization of dAtx2^NLS^. (D) Anti-dAtx2 immunoblot demonstrating similar expression levels of the different dAtx2 transgenes used in this work. The transgenes were driven by *gmr-GAL4.* Larval eye imaginal discs (ten per genotype) were used to avoid artifact potentially caused by cell degeneration or loss. dTubulin was blotted for loading control and larvae cultured at 25°C. Relative protein expression levels are indicated below normalized to the wild-type dAtx2 overexpressing line (dAtx2^OE^, underlined) (E–H) SEM images showing the eye phenotypes caused by the different dAtx2 transgenes at 25°C. Transgenes were expressed with *gmr-GAL4*. (E) Control eyes. (F) Wild-type dAtx2 over-expression (*dAtx2^OE^*) in the eye causes a mild phenotype with some ommatidial disorganization and bristle loss. (G) Expression of dAtx2^NES^ causes a similar phenotype to that caused by wild-type dAtx2. (H) Expression of dAtx2^NLS^ at similar levels as wild-type dAtx2 causes severe eye degeneration; the structure of the ommatidia is lost and interommatidial bristles are absent. In addition there is a strong decrease in eye size. Genotypes: (A, A′) *UAS-CD8-GFP/UAS-dAtx2[4]; ok107-GAL4/+*. (B, B′) *UAS-CD8-GFP/+; UAS-dAtx2^NES^/+; ok107-GAL4/+*. (C) *UAS-CD8-GFP/UAS-dAtx2^NLS^; ok107-GAL4/+*. (D–H) Control: *gmr-GAL4/UAS-GFP*. dAtx2^OE^: *gmr-GAL4/UAS-dAtx2[4]*. dAtx2^NLS^: *gmr-GAL4/ UAS-dAtx2^NLS^.* dAtx2^NES^: *gmr-GAL4/+; UAS-dAtx2^NES^ /+*.

The observations that expanded Ataxin-1 induces nuclear accumulation of Ataxin-2 and that decreasing the levels of endogenous Ataxin-2 suppresses toxicity in *SCA1^82Q^* flies, suggest that nuclear accumulation of Ataxin-2 may lead to neurotoxicity. To test this hypothesis, we generated dAtx2 transgenic flies with an exogenous nuclear localization signal (NLS) engineered on its C-terminus end (*dAtx2^NLS^*) (Figure 7 compare A-A′ with C). Although wild-type Ataxin-2 is only detected in the cytoplasm in *Drosophila* and human neurons, it is difficult to rule out the possibility that some Ataxin-2 may be present in the nucleus. Therefore, we also generated flies carrying a dAtx2 construct with an exogenous nuclear export signal (NES) (*dAtx2^NES^*) to use as an additional control (Figure 7B-B′). Ten transgenic lines were recovered for each dAtx2 construct, with a wide range of expression levels for each transgene. We selected transgenic lines expressing wild-type dAtx2 (dAtx2^OE^), dAtx2^NES^ or dAtx2^NLS^ at similar levels (Figure 7D compare lanes 2–4), and compared their toxicity in the eye. As shown in Figure 7E-H, dAtx2^NLS^ is more toxic than wild-type dAtx2 or dAtx2^NES^. While both wild-type dAtx2 and dAtx2^NES^ induce a relatively mild eye phenotype compared to controls (Figure 7 E-G), expression of dAtx2^NLS^ results in strong eye toxicity (Figure 7H). Eyes of dAtx2^NLS^ flies show a severely disorganized ommatidial lattice and a complete absence of interommatidial bristles (Figure 7H). These observations were consistent in several lines for each of the dAtx2 constructs at similar levels of expression (data not shown).

In summary, expression of dAtx2^NES^ and wild-type dAtx2 in the eye cause similar mild phenotypes. This is consistent with dAtx2 being mainly cytoplasmic, and with observations of SCA2 pathogenesis in the cytoplasm[44,45]. Interestingly, increasing the levels of Ataxin-2 in the nucleus is sufficient to cause a much more severe eye phenotype. These observations suggest that the toxicity of expanded Ataxin-1 is mediated in part, by the nuclear accumulation of Ataxin-2.

### Nuclear Ataxin-2 Decreases Sens Protein Levels and Induces Loss of Mechanoreceptor Bristles

**Figure 8:**
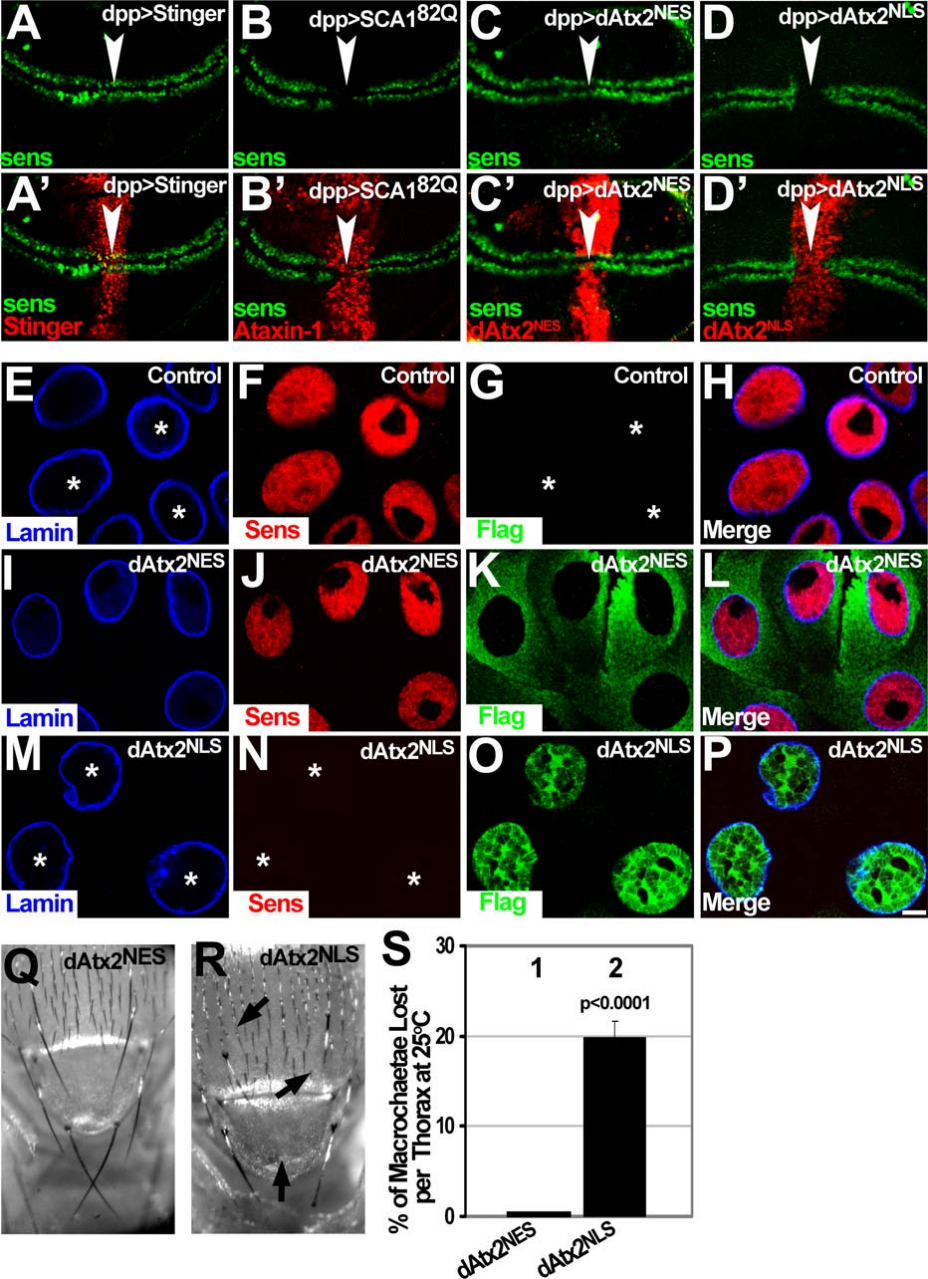
Nuclear dAtx2 Induces Decreased Levels of Senseless (Sens) Protein and Loss of Mechanoreceptors (A–D′) Immunofluorescence staining revealing Sens endogenous pattern in the wing margin SOPs on animals of genotypes indicated on top. (A and A′) Distribution of Sens in wing discs expressing a neutral NLS-DsRed protein (RedStinger) in the *dpp* territory (red in A′). Sens is detected as two parallel rows of cells (SOPs of the dorsal and ventral wing margins) uninterrupted in the area where they cross the *dpp* territory (arrowhead). (B, B′) Ataxin-1[82Q] eliminates Sens signal in the wing discs in a cell autonomous manner. Notice the gap in Sens signal when it crosses the *dpp* territory (arrowhead) expressing Ataxin-1[82Q] (red in B′). (C, C′) Expression of dAtx2^NES^ (red in C′) in the *dpp* territory does not affect Sens signal (arrowhead). (D, D′) Expression of dAtx2^NLS^ (red in D′) in the *dpp* territory reduces Sens signal in the SOPs, note the gap in Sens pattern (arrowhead). (E–P) Immunofluorescence staining revealing Sens (red, anti-Sens) and the dAtx2^NES^ or dAtx2^NLS^ proteins (green, anti-Flag), in salivary gland cells of the indicated genotypes. Nuclei are visualized using anti-Lamin (blue). (E–H) Control salivary gland cells showing robust Sens signal. Asterisks in G indicate the position of the nuclei. (I–L) dAtx2^NES^-expressing salivary gland cells have normal Sens levels when compared to controls. (M–P) Expression of dAtx2^NLS^ in salivary gland cells causes a decrease of Sens protein. Sens signal (red) is dramatically decreased in dAtx2^NLS^-expressing cells (compare N with F and J). Asterisks in N indicate the position of the nuclei. (Q–S) Effect of dAtx2^NES^ or dAtx2^NLS^ on adult thoracic macrochaetae formation after SOP specific expression. (Q) SOP-specific expression of dAtx2^NES^ causes no macrochaetae loss. (R) Expression of dAtx2^NLS^ in the SOP cells causes significant macrochaetae loss in the same conditions as Q. Arrows point out missing macrochaetae. (S) Quantification of lost macrochaetae in adult thoraxes of the same genotypes as Q and R. Column-1 shows no decrease in the number of macrochaetae per thorax in flies expressing dAtx2^NES^. Column-2 reveals increased loss of macrochaetae following SOP specific expression of Atx2^NLS^ (p<0.0001). 20 animals were used per genotype; data was analyzed using Students t; error bars represent s.e.m. Experiments in Q–S were performed at 25°C. Genotypes: (A, A′) *dpp-GAL4/ UAS-RedStinger*. (B, B′) *UAS-SCA1^82Q^[F7]/+; +;dpp-GAL4/+*. (C, C′, I–L) *dpp-GAL4/UAS-dAtx2^NES^*. (D, D′, M–P) *UAS-dAtx2^NLS^* /+; *dpp-GAL4/+* (E–H) *dpp-GAL4/+*. (Q–S) dAtx2^NES^: *sca^109–68^-GAL4/+; UAS-dAtx2^NES^ /+*. dAtx2^NLS^: *sca^109–68^-GAL4/ UAS-dAtx2^NLS^*.

To further test the hypothesis that nuclear Ataxin-2 contributes to expanded Ataxin-1-induced toxicity, we analyzed the effect of expressing the different *dAtx2* transgenes on Sens distribution in the wing margin SOPs. Expression of Ataxin-1[82Q] in the antero-posterior compartment boundary (using *dpp-GAL4*) induces a cell autonomous decrease of Sens only in the *dpp-GAL4* expressing area of the wing margin (Figure 8A, A′ and 8B, B′, arrowhead). This provides a molecular readout of Ataxin-1[82Q]-induced neurotoxicity.

Expression of dAtx2^NES^ from *dpp-GAL4* does not reduce the levels of Sens, whose distribution in imaginal wing discs is unchanged (Figure 8C and C′, arrowhead). Next, we tested the effect of dAtx2 with a nuclear localization signal (*dAtx2^NLS^*). Like Ataxin-1[82Q], expression of dAtx2^NLS^ induces loss of Sens in the wing margin SOPs (Figure 8D and D′, arrowhead). dAtx2^NLS^-induced loss of Sens is also observed in other cell types. Salivary gland cells express Sens at high levels [20], which localizes to the nucleus (Figure 8E-H). Salivary gland cells expressing dAtx2^NES^ show no detectable change in Sens levels or distribution (Figure 8I-L). In contrast, expression of dAtx2^NLS^ causes a dramatic decrease in the amount of Sens (Figure 8M-P). The nuclei of *dAtx2^NLS^* cells are still present and their morphology is similar to controls, indicating that Sens loss is unlikely a consequence of cell death. Therefore, nuclear dAtx2 mimics Ataxin-1[82Q] in causing loss of Sens protein accumulation.

Expression of Ataxin-1 in the thoracic SOPs (using *sca-GAL4*) leads to loss of macrochaetae in the adult thorax (Figure 3 and ref.[18]). Therefore, we also investigated the consequences of nuclear or cytoplasmic Ataxin-2 accumulation on the development of adult macrochaetae. As with wild-type dAtx2 (Figure 3), expression of dAtx2^NES^ in the thoracic SOP cells causes no visible change in the number of macrochaetae in the adult thorax (Figure 8Q and Figure 8S column-1). In contrast, expression of dAtx2^NLS^ induces a significant decrease in the number of macrochaetae, with an approximate twenty percent reduction in comparison to control animals (Figure 8R and Figure 8S column-2). These results indicate that increasing the levels of nuclear dAtx2 mimics expanded Ataxin-1 in inducing loss of mechanoreceptors and reducing the levels of Sens protein.

## Discussion

Here we report functional interactions between the proteins causing two distinct Spinocerebellar ataxias. We use a *Drosophila* model of SCA1 to show that wild-type dAtx2 (the fly homolog of the protein that when expanded causes SCA2) mediates, at least in part, neuronal degeneration caused by expanded Ataxin-1 (the protein triggering SCA1). Ataxin-1[82Q]-induced toxicity is worsened by increasing the levels of dAtx2. More significantly, decreasing the levels of dAtx2 suppresses expanded Ataxin-1-induced neuronal degeneration as shown in several independent assays. The suppression of Ataxin-1[82Q] phenotypes by partial loss of function of *dAtx2* argues against a possible mechanism by which sequestration and depletion of Ataxin-2 contributes to expanded Ataxin-1-induced neurodegeneration. This is further supported by lack of cerebellar or other neuronal abnormalities in mice that are deficient for Ataxin-2[46].

We find that the human expanded Ataxin-1 interacts with the dAtx2 and human Ataxin-2 proteins in co-AP assays. Furthermore, overexpressed Ataxin-1 pulls down endogenous hAtaxin-2 in cultured cells. These results suggest that Ataxin-1 and Ataxin-2 may be functional interactors in vivo. Consistent with this, we find that expanded Ataxin-1 induces accumulation of Ataxin-2 in the nucleus, where the two proteins localize in NIs both in *Drosophila* neurons and SCA1 human brain tissue. These are surprising observations since Ataxin-2 is normally a cytoplasmic protein both in humans and *Drosophila*. Interestingly, wild-type Ataxin-1 can cause neurotoxicity when overexpressed, although to a much lesser extent than expanded Ataxin-1 [25]. However, nuclear accumulation of dAtx2 is triggered by pathogenic but not wild-type forms of Ataxin-1, at least in detectable amounts. Taken together these data suggested that accumulation of Ataxin-2 in the nucleus contributes to the exacerbated toxicity of expanded Ataxin-1, and is an important mechanism of pathogenesis in SCA1. To investigate this hypothesis we targeted dAtx2 to the nucleus by means of an exogenous NLS signal. We find that dAtx2^NLS^ is sufficient to cause a dramatic increase of its toxicity, when compared to either wild-type dAtx2 or dAtx2 with an exogenous nuclear export signal (dAtx2^NES^) expressed at similar levels.

To further test the hypothesis that nuclear accumulation of Ataxin-2 contributes to neurodegeneration caused by expanded Ataxin-1 we investigated Sens levels. Sens and its murine orthologue Gfi1 are proneural factors whose levels are decreased in the presence of expanded Ataxin-1[18]; thus providing a molecular readout for the neurotoxicity of Ataxin-1. In *Drosophila*, reduction of Sens levels leads to the loss of mechanoreceptors [18], so we monitored Sens in the context of flies expressing either dAtx2^NLS^ or dAtx2^NES^ but not carrying the Ataxin-1[82Q] transgene. We find that nuclear targeted, but not cytoplasmic, dAtx2 mimics both the Sens reduction and mechanoreceptor loss phenotypes caused by Ataxin-1[82Q].

Expanded Ataxin-2 accumulates both in the cytoplasm and the nuclei of SCA2 postmortem brains [2,47–49]. In mouse and cell culture models of SCA2, expanded Ataxin-2 accumulates in the cytoplasm and its nuclear accumulation is not necessary to induce toxicity [44,45]. However, nuclear accumulation of expanded Ataxin-2 also occurs in cultured cells [45], and is consistently observed in human SCA2 postmortem brainstem neurons [2,47–49]. These observations suggest that both nuclear and cytoplasmic mechanisms of pathogenesis contribute to neurodegeneration in SCA2, as it is known to occur in other polyglutamine diseases like HD and SCA3 [50–52]. One possibility is that Ataxin-2 shuttles between the nucleus and the cytoplasm although the protein is normally detected only in the cytoplasm. Our data show that accumulation of dAtx2 in the nucleus is more harmful than in the cytoplasm. Thus, neurons with nuclear Ataxin-2 in SCA2 patients may be relatively more compromised than neurons where Ataxin-2 accumulates in the cytoplasm. In agreement with this possibility, expanded Ataxin-2 is found in the nuclei of pontine neurons of SCA2 brains, one of the neuronal groups and brain regions with prominent degeneration in SCA2 [2,47–49].

Reducing Ataxin-2 levels suppresses expanded Ataxin-1 toxicity, strongly arguing against a mechanism of pathogenesis by loss of function of Ataxin-2 in the cytoplasm. Studies of the normal function of Ataxin-2 and its yeast [29,30], C. elegans [31], and *Drosophila* [32,33] homologs suggest a role in translational regulation. Thus, an attractive possibility is that Ataxin-1 [82Q] requires dAtx2 to impair Sens translation and induce the loss of mechanoreceptors. Consistent with this hypothesis is the finding that partial loss of function of dAtx2 suppresses the loss of mechanoreceptors phenotype caused by expanded Ataxin-1.

The data described here uncover unexpected functional interactions between proteins involved in two different SCAs. Nuclear accumulation of Ataxin-2, normally a cytoplasmic protein, is a common denominator of SCA1 and SCA2, and leads to reduced levels of at least one important proneural factor; i.e. Sens, whose mammalian orthologue Gfi1 is required for Purkinje cell survival [18]. Thus neuronal degeneration may take place through common mechanisms in different ataxias, and one of these mechanisms may involve the abnormal accumulation of Ataxin-2 in neuronal nuclei.

## Methods

### Drosophila strains.

The cDNA GH27029 containing *dAtx2* was obtained from the BDGP repository. SV40 nuclear localization signal and PKI nuclear export signal were engineered on the 3′ end of *dAtx2* cDNA by PCR. Both constructs were then subcloned first in pGEM^®^-T (Promega) and then in a previously generated pUAST-flag expression vector[53,54]. The *Drosophila* transgenic lines *UAS-dAtx2^NES^* and *UAS-dAtx2^NLS^* were obtained by injecting both constructs following standard procedures. *EP(3)3145* was obtained from the Szeged Drosophila Stock Center in Hungary. The wild type UAS-dAtx2 (dAtx2OE) and mutant dAtx2-X1 lines [33] were kindly provided by Dr. Pallanck, L.J.. The UAS-SCA182Q, UAS-SCA130Q and UAS-SCA12Q lines have been previously described [7,25]. N-Htt128Q flies have been previously described [7,43]. All other Drosophila strains were obtained from the Bloomington Drosophila Stock Center at Indiana University.

### Scanning electron microscopy and retinal paraffin sections.

We used previously published procedures [7,25].

### Scoring of macrochaetae in adult thoraxes.

Flies were raised at either 25°C for low Ataxin-1[82Q] expression levels or 27°C for high Ataxin-1[82Q] expression levels. Flies were collected at day 1 and the number of macrochaetae per thorax of same sex flies was counted for 20 animals per genotype. The percentage of lost macrochaetae over a total of 26 was calculated, and the average per genotype was plotted in the chart.

### Motor performance assay.

Between 25 and 30 adult females per genotype are collected for periods no longer than 24 hours. Flies are transferred to vials containing new food every day. The assay is carried out in an empty vial. The vial is tapped so all flies fall to the bottom then we score flies that climb past a line 5cm high in 18 seconds, and this procedure is repeated ten times for each day shown in the chart. The average percentage of flies climbing per day is then calculated and plotted in Microsoft Excel. Experiments are always performed at the same time in the day to ensure no circadian rhythm effects. Four replicas (25<n<30) are analyzed per genotype. Motor performance in each replica is measured until all animals failed to reach the 5 cm line.

### Protein–protein interaction assay.

Gst co-affinity purification assays between GST-ATXN1^2Q^, GST-ATXN1^30Q^, GST-ATXN1^82Q^, GST-ATXN1N-term^82Q^, GST-ATXN1C-term GST-ATXN1-AXH or GST-ATXN1^82Q^S776A, and dAtx2, Flag-dAtx2 or Myc-hAtaxin-2 were carried out as previously published [55]. pEGFP-C1 vector was used for control and constructs were transfected in HEK293T cells. Immunoblots were carried out following standard procedures and stained with mouse anti-Myc (9E10) and rabbit anti-GST antibodies (Sigma).

### Immunofluorescence staining.

Tissues were dissected and fixed in 4% formaldehyde. Following standard procedures tissues were incubated with rabbit anti-dAtx2 (1:2000, courtesy of Leo J. Pallanck), Lc2628 anti-nuclear Lamin (1:50, Hybridoma Bank), rabbit anti-flag (1:200, Sigma), 11NQ rabbit anti-Ataxin-1 (1:750), 6C1 mouse anti-Ubiquitin (Sigma), guinea pig anti-Sens (1:1000, Hugo Bellen). Secondary antibodies were obtained from Jackson Labs and Molecular Probes. The fluorescent images were documented in a LSM510 Zeiss confocal microscope.

### Quantification of Sens signal.

Sens quantification in the SOP and bristle precursor cells of the wing margins was carried as follows: animals were raised at 25°C. immunofluorescence was done on wing discs of the different genotypes (gp anti-Sens 1:1000, Hugo Bellen). Confocal images were obtained throughout the thickness of the wing margin (20 sections per wing disc with 2 μm interval) and then the signal was stacked and summed. Quantification was carried out by selecting the area covered by each wing margin (dorsal or ventral) separately and calculating fluorescence and area. Data corresponding to 20 wing discs per genotype was analyzed by Tukey-Kramer HSD and plotted in a chart.

### Immunohistochemistry on human post-mortem brain tissue.

Materials were stained as previously described [7].

### dAtx2 immunoblotting.

Eye imaginal discs from ten larvae per genotype were dissected in cold PBS, homogenized in 30 μl of Laemmli buffer (Bio-Rad) using a pellet pestle motor (Kontes) and loaded on a 7.5% Tris-HCl Ready Gel (Bio-Rad). Membranes were stained with primary antibodies rabbit anti-dAtx2 (1:5000, L.J. Pallanck) and mouse anti-tubulin (Hybridoma Bank, 1:1000). Horseradish peroxidase-conjugated anti-mouse or anti-rabbit IgG secondary antibodies (1:5000; Bio-Rad) were used and membranes were developed using ECL Western blot detection kit (Amersham Biosciences).

## Supporting Information

Figure S1 Co-Expression of dAtx2 Reverts the Suppression of Ataxin-1 [82Q]-Induced Degeneration Observed in *SCA1^82Q^/dAtx2^X1^* Animals (A-D′) SEM images of eyes from flies of the genotypes indicated on top. (A, A′) normal shape and arrangement of ommatidia in control animals (*w; gmr-GAL4/UAS-GFP; +*). (B, B′) Ataxin-1 [82Q] expression causes distortion of ommatidia and loss of inter-ommatidial bristles in *SCA1^82Q^* animals (*UAS-SCA1^82Q^/+; gmr-GAL4/+*). (C, C′) Suppression of the phenotype induced by Ataxin-1 [82Q] in flies with only one functional copy of dAtx2 (*UAS-SCA1^82Q^/+; gmr-GAL4/+; dAtx2^X1^/+*). Note in C′ improved arrangement of ommatidia, and more interommatidial bristles. (D, D′) Expression of low levels of dAtx2 in Ataxin-1 [82Q] flies that are also heterozygous for the *dAtx2^X1^* allele (*UAS-SCA1^82Q^/+; gmr-GAL4/UAS-dAtx2[B]; dAtx2^X1^/+*) reverts the suppression observed in *SCA1^82Q^/dAtx2^X1^*flies. Note that the eye phenotype observed in D and D′ is more similar to B and B′ than to C and C′ both at the level of ommatidial disorganization, as well as bristle loss.
(5.7 MB TIF)

Figure S2 Levels of dAtx2 Protein in Control and Mutant Flies Carrying the Alleles Used in This Work (A) Comparison between dAtx2 in wild-type and homozygous mutant *dAtx2^X1^/dAtx2^X1^* ovaries. Note the absence of dAtx2 signal in *dAtx2^X1^/dAtx2^X1^* ovaries.
(B) Increased levels of dAtx2 are observed when dAtx2 is overexpressed in the eye with the *dAtx2^OE^* line, in comparison with the levels observed in wild-type flies.
Genotypes: (A) Control: *yw.* *dAtx2^X1/X1^*:*HS-Flp/+; FRT, dAtx2^X1^/ FRT, dAtx2^X1^*. (B) Control: *w; gmr-GAL4/+; +.* dAtx2^OE^: *w; gmr-GAL4/UAS-dAtx2;+*.
(3.3 MB TIF)

### Accession Numbers

NCBI protein accession data: NP_002964, NP_732034, NP_650466, NP_732033, NP_524818, NP_002102, NP_000323; OMIM disease reference: #164400, #183090.
