# Supplementary figures and images for: dAtaxin-2 Mediates Expanded Ataxin-1-Induced Neurodegeneration in a Drosophila Model of SCA1
Source: PLoS Genet. 2007 Dec 28;3(12):e234. doi: 10.1371/journal.pgen.0030234 (PMC2323314; doi:10.1371/journal.pgen.0030234)

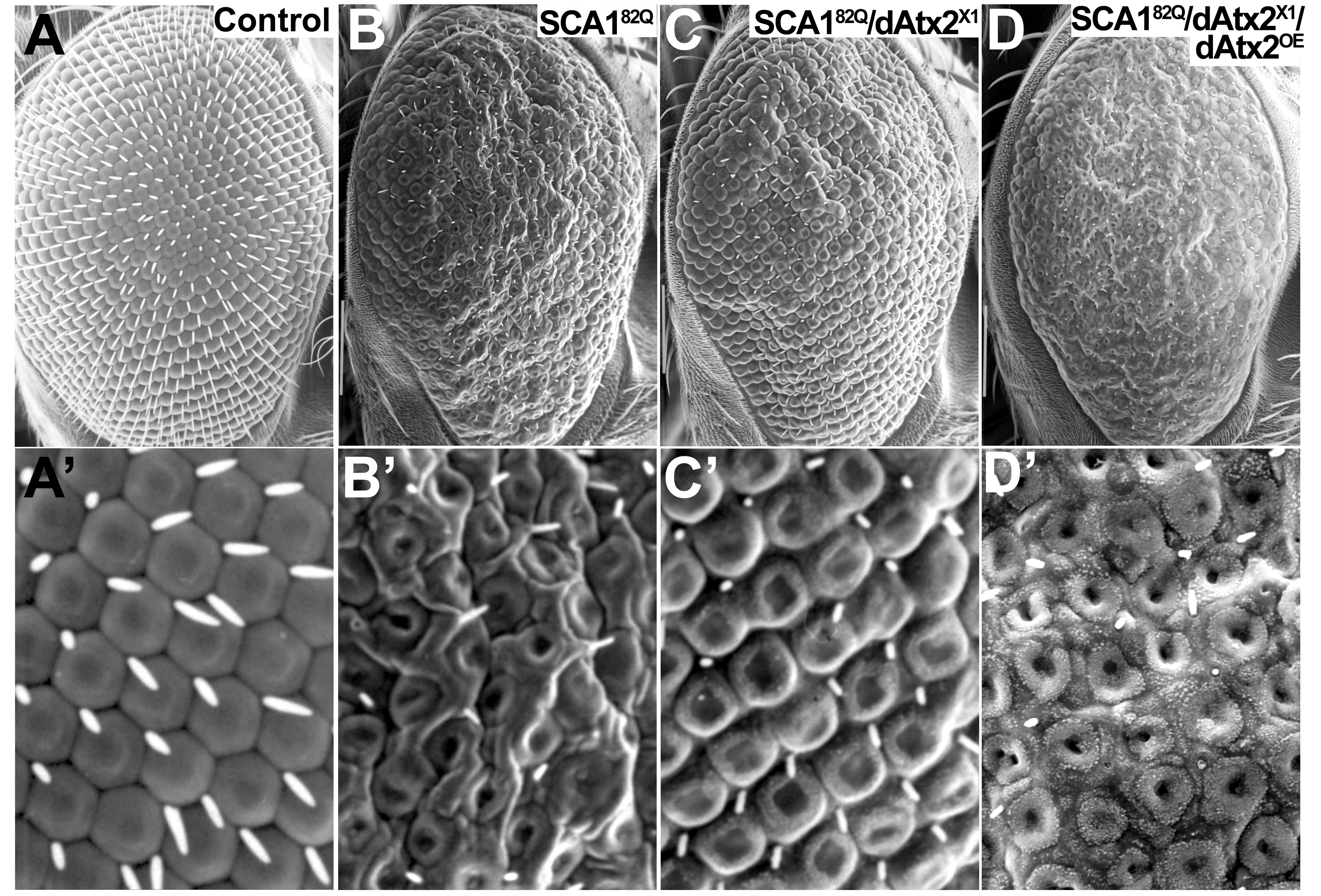

Supplement: Figure S1 — (A-D′) SEM images of eyes from flies of the genotypes indicated on top. (A, A′) normal shape and arrangement of ommatidia in control animals (w; gmr-GAL4/UAS-GFP; +). (B, B′) Ataxin-1 [82Q] expression causes distortion of ommatidia and loss of inter-ommatidial bristles in SCA182Q animals (UAS-SCA182Q/+; gmr-GAL4/+). (C, C′) Suppression of the phenotype induced by Ataxin-1 [82Q] in flies with only one functional copy of dAtx2 (UAS-SCA182Q/+; gmr-GAL4/+; dAtx2X1/+). Note in C′ improved arrangement of ommatidia, and more interommatidial bristles. (D, D′) Expression of low levels of dAtx2 in Ataxin-1 [82Q] flies that are also heterozygous for the dAtx2X1 allele (UAS-SCA182Q/+; gmr-GAL4/UAS-dAtx2[B]; dAtx2X1/+) reverts the suppression observed in SCA182Q/dAtx2X1flies. Note that the eye phenotype observed in D and D′ is more similar to B and B′ than to C and C′ both at the level of ommatidial disorganization, as well as bristle loss. (5.7 MB TIF) [file pgen.0030234.sg001.tif]

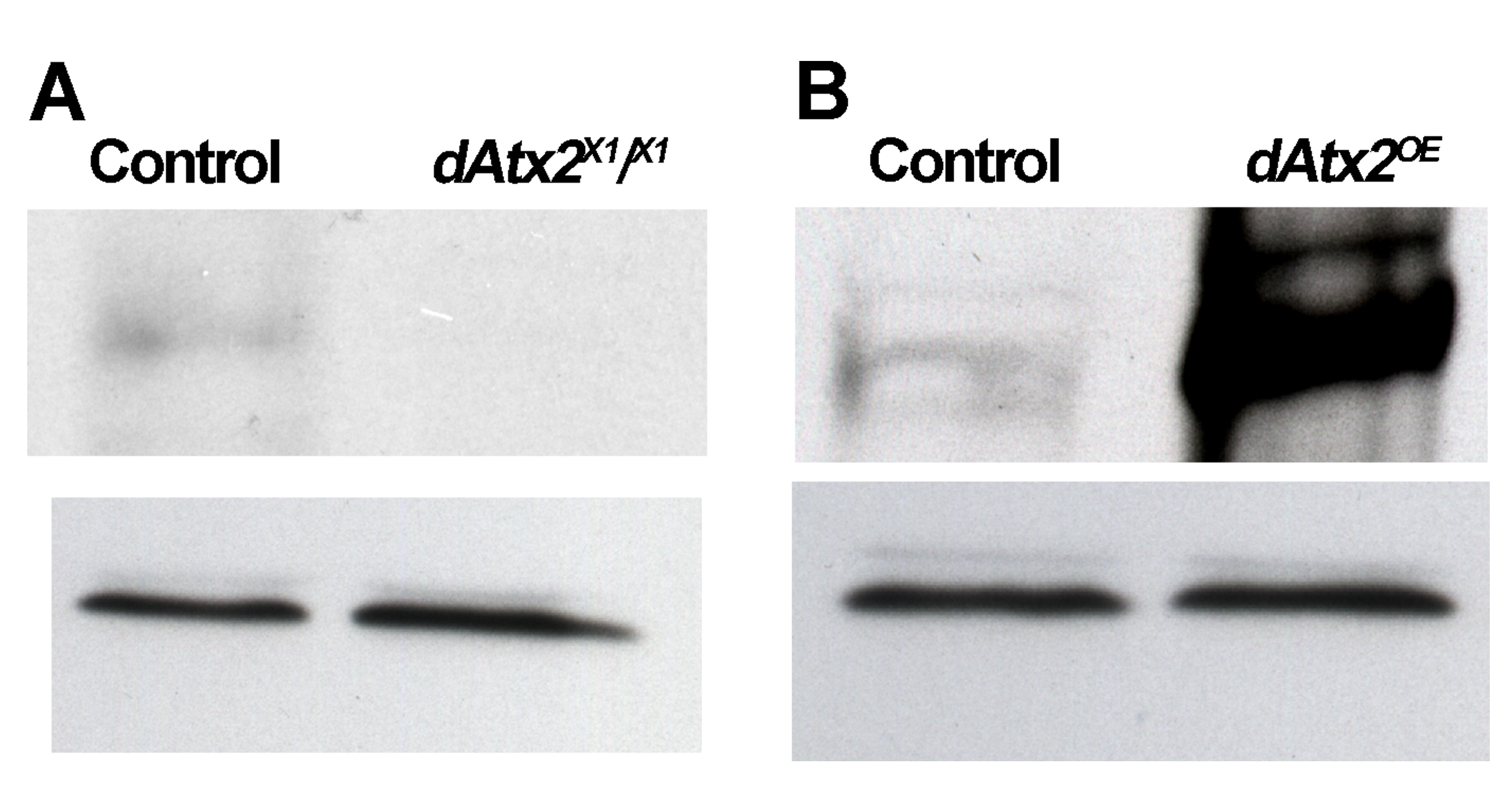

Supplement: Figure S2 — (A) Comparison between dAtx2 in wild-type and homozygous mutant dAtx2X1/dAtx2X1 ovaries. Note the absence of dAtx2 signal in dAtx2X1/dAtx2X1 ovaries. (B) Increased levels of dAtx2 are observed when dAtx2 is overexpressed in the eye with the dAtx2OE line, in comparison with the levels observed in wild-type flies. Genotypes: (A) Control: yw. dAtx2X1/X1:HS-Flp/+; FRT, dAtx2X1/ FRT, dAtx2X1. (B) Control: w; gmr-GAL4/+; +. dAtx2OE: w; gmr-GAL4/UAS-dAtx2;+. (3.3 MB TIF) [file pgen.0030234.sg002.tif]
